# Building a Hierarchical Organization of Protein Complexes Out of Protein Association Data

**DOI:** 10.1371/journal.pone.0100098

**Published:** 2014-06-30

**Authors:** Aleksandar Stojmirović, Yi-Kuo Yu

**Affiliations:** National Center for Biotechnology Information, National Library of Medicine, National Institutes of Health, Bethesda, Maryland, United States; Koc University, Turkey

## Abstract

Organizing experimentally determined protein associations as a hierarchy can be a good approach to elucidating the content of protein complexes and the modularity of subcomplexes. Several challenges exist. First, intrinsically sticky proteins, such as chaperones, are often falsely assigned to many functionally unrelated complexes. Second, the reported collections of proteins may not be true “complexes” in the sense that they bind together and perform a joint cellular function. Third, due to imperfect sensitivity of protein detection methods, both false positive and false negative assignments of a protein to complexes may occur. We mitigate the first issue by down-weighting sticky proteins by their occurrence frequencies. We approach the other two problems by merging nearly identical complexes and by constructing a directed acyclic graph (DAG) based on the relationship of partial inclusion. The constructed DAG, within which smaller complexes form parts of the larger, can reveal how different complexes are joined. By merging almost identical complexes one can deemphasize the influence of false positives, while allowing false negatives to be rescued by other nearly identical association data. We investigate several protein weighting schemes and compare their corresponding DAGs using yeast and human complexes. We find that the scheme incorporating weights based on information flow in the network of direct protein–protein interactions produces biologically most meaningful DAGs. In either yeast or human, isolated nodes form a large proportion of the final hierarchy. While most connected components encompass very few nodes, the largest one for each species contains a sizable portion of all nodes. By considering examples of subgraphs composed of nodes containing a specified protein, we illustrate that the graphs' topological features can correctly suggest the biological roles of protein complexes. The input data, final results and the source code are available at ftp://ftp.ncbi.nlm.nih.gov/pub/qmbpmn/ProteinComplexDAG/.

## Introduction

A living cell functions through coordination of its molecular components, within which proteins play a key role. In many cases proteins do not act individually but by joining with other proteins to form functional complexes consisting of several identical or different subunits. The last decade has seen a surge of interest in generating networks of interactions between cellular proteins in a variety of organisms [1]–[3]. Such interest resulted in development of a large variety of techniques, both experimental and computational, for assessing which proteins interact with each other within a cell and under what circumstances. Generally, the experimental techniques can be divided in two complementary classes. The first includes the methods such as yeast-two-hybrid [4], [5] and fluorescence resonance energy transfer (FRET) [6], which determine whether two individual proteins can interact and therefore report *binary* interactions. The second class of methods isolate groups of proteins from cellular mixtures and therefore report protein *associations*, without necessarily providing the information about which proteins directly interact within the mixture. These techniques may be based on fractionations of cellular extracts using liquid chromatography [7], [8], or on affinity-based pull-downs thorough immunoprecipitation [9] or tandem affinity purification [10], [11]. While these isolation techniques have a disadvantage that they do not reveal the exact binding patterns of proteins, they offer more information than binary interactions by associating several proteins together.

The results of more than a decade of intensive investigations of protein interactions in model organisms can be easily retrieved. Numerous existing public databases, such as BIND [12], [13], BioGRID [14], CORUM [15], DIP [16], HPRD [17], IntAct [18], MINT [19], MPact [20], MPPI [21] and OPHID [22], store that information and curate publications. The interactions reported come both from large, systematic high-throughput studies and from curations of publications focusing on particular cellular subsystems. Thanks to the iRefIndex database [23], which agglomerates the information from all the aforementioned public databases in a easily accessible way, it is now possible to perform systematic analyses of almost all evidences for interactions and complexes.

In this paper we present a novel way to organize protein association data, aiming to elucidate structure of protein complexes and modularity of subcomplexes (which may be interchangeable). It is well known that proteins or complexes can be multifunctional [24], that is, perform very different functions depending on the context in which they appear. To elucidate the structural relationships between various protein complexes we propose to construct a hierarchy, where smaller complexes form parts of the larger. To account for the possibilities that a complex may serve as a subunit of several structurally and functionally distinct larger complexes, our hierarchy takes the form of a directed acyclic graph (DAG). The absence of cycles (acyclic), implying that no node can reach itself after following several directed edges in the graph, naturally prevents two distinct complexes from being the subunit of each other. Another desirable feature of a DAG is that a child node (or a subunit) may point to multiple *unrelated* parent nodes (larger complexes). This is in contrast to previous tree-resulting approaches, such as [25], that only allow one immediate parent node for each child node considered. The edges of our DAG are based on the relationship of partial inclusion: 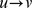 (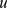 embeds into 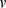) in the DAG implies that 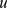 is a part of 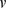 but not vice versa. Since our goal is to reconcile the unprocessed primary results, we only include the interaction and association evidences that were experimentally obtained. It is worth pointing out that another recent work [26], although also resulting in a DAG, takes a very different route. It starts by constructing a binary tree, then introduces heuristics to allow modification of the tree structure to allow for more than two children nodes per parent, followed by another heuristic to allow a child node to link to multiple parent nodes to form a DAG.

There are several challenges associated with building a hierarchy of protein complexes based on experimental findings. The first challenge is what to do with the sticky proteins? Due to their inherent trend to bind other proteins, *intrinsically* sticky proteins may be falsely assigned to many groups of functionally organized but unrelated complexes. Second, the reported collections of proteins may not be true ‘complexes’ in the sense that they bind together and perform a joint cellular function. For example, it is possible that the prey proteins retrieved using one bait may belong to several independent complexes. Hence, the assemblies derived from interaction databases should be properly called ‘associations’ (although we will for simplicity call them complexes). Third, due to imperfect sensitivity of protein detection methods, it is possible to obtain both false positive and false negative assignments of a protein within a particular association. We mitigate the first issue by down-weighting these sticky proteins according to their numbers of associated complexes. To avoid accidentally up-weighting *infrequent* proteins that only appear in a very small number of associations, we set a lower bound on the number of complexes each protein participates in. We tame the other two problems by constructing a DAG and by merging nearly identical complexes. The constructed DAG can show how different complexes are joined by a common bait protein. By merging almost identical complexes one can down-weight false positives while at the same time allow false negatives in one association data to be rescued by other nearly identical association data.

## Methods

### Overview

We construct DAGs for protein complexes in yeast and human, the two species with the most abundant association and interaction data. Each node in the hierarchy represents a protein complex and each edge a sub-part relationship. A directed edge links a complex 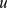 to a complex 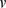 if 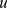 can be considered a part or a subunit of 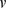. Each complex is supported by one or more experimental evidences and represented by a weight function that assigns positive weights for all its member proteins and zero to all other proteins. The part-of relationship is established by first comparing the weight functions by similarity and then linking most similar complexes.

### Datasets of protein complexes and binary interactions

We obtained the evidences for protein complexes (associations) and protein-protein interactions in yeast (*Saccharomyces cerevisiae*) and human (*Homo sapiens*) by running the ppiTrim [27] script (Version 1.3) with the iRefIndex [23] database (Candidate for Release 10.0, dated Nov 23rd 2012) as input (see Text S1 and Figure S1). The ppiTrim script outputs evidences for three classes of interactions: directed binary interactions (biochemical reactions with asymmetric biological roles of participants), undirected binary interactions (physical bindings), and protein complexes (associations of more than two proteins). Only the latter two categories were used since the current study targets on physical bindings/associations. Each evidence is associated with two or more protein interactors, a source publication, and multiple annotations including interaction detection method, interaction type and experimental roles of interactors (bait/prey). An evidence may originate from a record in a single source database or be an agglomeration of several records from different sources. For each investigated species, the output of ppiTrim was processed into a set of direct binary interactions (PPI set) and a set of protein complexes (PC set). The PPI set was obtained by selecting all binary interaction evidences annotated with interaction type ‘direct interaction’ and grouping them by their protein interactors. All interactions of a protein with itself were removed. The PC set was constructed from all evidences for protein complexes with more than three different proteins. To avoid apparent redundancies, complex records sharing the same publication, experimental annotation and bait protein were grouped together and considered as a single complex. In addition to the complexes available through ppiTrim, we also included in the PC set the human complexes reported by Havugimana *et al.* [28], which were not available in iRefIndex at the time we processed it. We took all complexes of size greater than 3 reported by Havuginama *et al.* and mapped their protein identifiers to Entrez Gene IDs using the algorithm employed by ppiTrim. We excluded the interactions from the Collins *et al.* paper [25] (in yeast), and from the OPHID [22] database since they were obtained by computational analysis of several primary datasets and hence do not represent direct experimental evidences.

### Protein Weights

Let 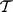 denote the set of all proteins for a given species. An *evidence* for a protein complex is a set of proteins specified with a record in the PC set. To account for its redundancy, an evidence 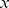 is associated with a weight 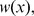 which takes the value 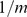 when this evidence and other 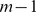 evidences share the same publication, experimental method annotation and bait protein. When this happens, these 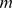 evidences are called *equivalent*. However, due to differences in curation, equivalent evidences may still be different, meaning that they can have different protein lists. For this reason and because we intend to merge highly similar complex nodes (as described later), a *protein complex node* 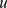 in our formulation is generally associated with a set of evidences 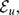 not just a single evidence. Each protein complex node 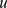 also has three weighting functions 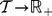 that assign weights to member proteins: support weights 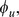 adjusted weights 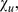 and information flow weights 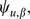 which depend on an additional parameter 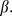 We denote by
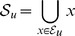
the set of of all protein *members* (or supports) of 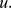 Each weighting function associated with 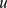 is positive on 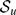 and zero outside of it. We will use the operator 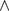 to denote the minimum 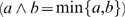 and 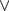 to denote the maximum (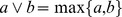). For a set 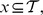 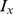 denotes the indicator function of 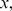 which takes the value 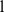 on 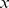 and 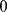 outside of it.

#### Support weights

The support weight function for a complex 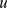, denoted 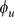, indicates the strength of evidence for membership in 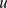 for each protein in 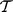. It only depends on the evidences associated with 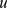:
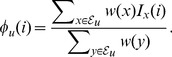
(1)


One can see that 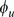 ranges between 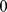 and 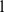; 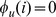 if protein 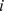 is not a member of any evidence in 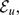 while 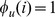 if all evidences contain 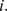


#### Adjusted weights

The adjusted weight function for a complex 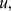 denoted 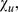 is similar to 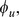 but penalizes proteins that are overrepresented in the PC set. Let 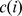 denote the (weighted) number of evidences containing protein 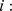


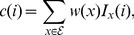
(2)where 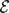 is the set of all evidences from the PC set. Then,
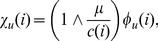
(3)where 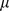 is the weighted median of 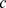. More precisely, let 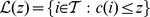. Then, 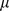 is selected as the largest value of 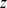 such that



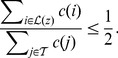
(4)Hence, for proteins that are infrequent and contribute cumulatively less than a half of the ‘mass’ of all complexes, their 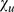 values equal their 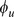 values. The weight of each of the rest of the proteins is proportionally reduced by the ratio 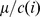.

#### Information flow weights

Information flow weights generalize adjusted weights by taking into account the direct protein-protein interactions within complexes. We represent the PPI set 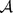 as a symmetric adjacency operator (matrix) 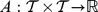, where
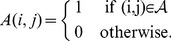
(5)


For each protein 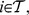 let 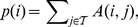 the total number of proteins interacting with 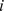 in the PPI set, and let 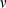 be the median value of 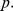 Construct a stochastic transition matrix 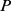 with matrix elements
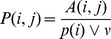
(6)and define the operator 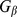 by
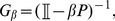
(7)where 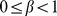 and 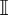 denotes the identity operator. Since the matrix 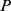 is stochastic, 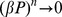 as 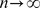. Thus the operator 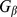 is well defined. For a complex 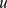 and protein 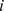, let
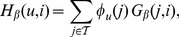
(8)and let



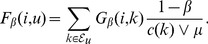
(9)Then, the information flow weight function 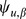 is defined by
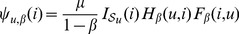
(10)




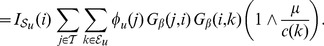
(11)


**Figure 1:**
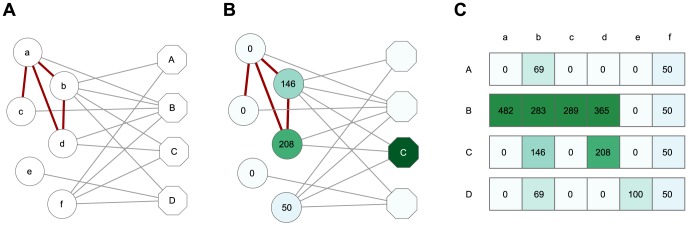
Obtaining information flow weights. We first construct a protein-complex graph where each complex is linked to its members (**A**). The hexagonal nodes labelled with upper case letters here denote complexes, while the circular nodes labelled with lower case letters denote proteins. The grey edges represent memberships of proteins within complexes, while red edges are direct protein-protein interactions. For each protein member of a given complex (complex C used here as an example), we evaluate its number of visits under our model (**B**). The numbers of visits (multiplied by 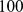) are shown on the protein nodes and also indicated by color (darker means larger values). The computed numbers of visits for all proteins constitute the ITM weight functions (**C**), which are used to construct the DAG. The values are multiplied by 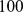. This example uses 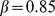 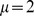 and 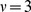. Note that the weights for proteins b and d are smaller in the complex C than in B, because B also includes their direct interaction partner a. Also observe that the proteins b and f are downweighted because they belong to 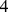 complexes while 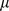 is set to 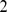.

Information flow weights can be interpreted in the context of the channel model of network information flow from [29]. Conceptually, we form an undirected network consisting of two types of nodes: complexes and proteins (Fig. 1). Each protein in the network is linked to each complex it belongs to, as well as to each of its interaction partners in the PPI set. To characterize a complex 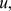 we place random walkers on the network nodes corresponding to its protein members, and allow each walker to wander through the network in discrete steps. The number of walkers initially placed on the protein node 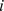 is proportional to 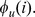 At each step, a walker either moves to another protein node with the probability 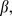 or moves to a possibly different complex node with the probability 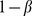 and terminates. Since a random walk is a stochastic process, and each walk proceeds independently, we evaluate the cumulative behavior of infinitely many walkers following the same rules. The operator 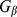 is known as the Green's function or the fundamental matrix [30]. For two protein nodes 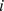 and 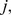 the value of 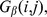 provides the expected number of visits to 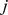 by a random walker originating at 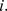 Hence, the value of 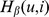 gives the expected number of visits of the protein node 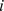 by all random walkers associated with 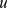 before they terminate [31].

Each walker visits at least one member of 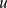: its initial point. Depending on the direct protein-protein links and the parameter 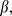 it may visit additional members of 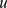 or move outside of 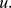 To ensure that only the visits of the walkers that terminate at the 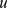-node count towards the final weights of proteins, 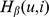 is multiplied by 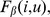 which can be interpreted as the probability that a walker at 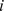 eventually terminates at 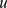 [29], [31]. To do so, the walker must first reach a node 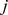 corresponding to a protein member of 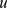 and then jump to 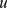. The probability of this jump is 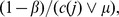 where the expression 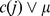 gives the adjusted number of complexes containing 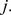 To avoid the situation where the proteins present in very few complexes carry excessive weights, all proteins are assumed to belong to at least 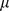 complexes. In a similar fashion, the parameter 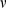 ensures that the probability of a protein-to-protein jump is at most 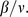 Therefore, the expression 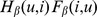 in (10) evaluates the expected number of walkers, originating at the members of 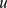 and terminating at the 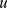-node in the adjusted protein-complex network, that visit protein node 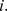 The 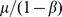 factor in (10) ensures that 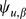 and 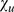 have the same scale, while 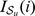 sets the value for all non-members of 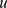 to 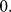


Since 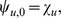 the adjusted weights can be interpreted as information flow weights without the contribution of the PPI set. In general, the value of 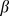 indicates the relative importance of direct binary interaction evidences from the PPI set compared to evidences for complexes from the PC set. For small 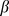, the final information flow weights are close to adjusted weights. When 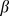 is large (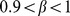), the final weights mostly depend on the direct protein-protein interactions. The value of 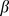 can be related to the average time 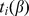 that a random walker originating at 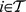 spends in the network before terminating [30]:
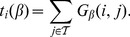
(12)


One can easily show that 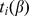 monotonically increases for 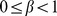 and that for 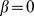 only the initial node is visited before termination so that 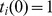 for every protein 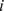. To facilitate consistent interpretation of the information flow weights in different species, we set the value of 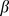 to correspond to a specified average time 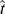 of the walk for a walker randomly placed at an initial protein node. To do so, we define 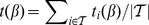 and solve the equation 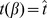 for 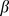 using Newton's method.

### Construction of a hierarchy

Here we describe the procedure for constructing a hierarchy that treats a protein weight function as an input parameter. Each protein weighting scheme results in a different final hierarchy.

#### Similarity measure between complexes

For a complex 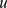, let 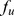 denote an arbitrary weight function associated with 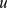 (i.e. either 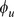, 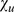 or 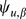). The similarity of two complexes 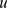 and 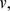 denoted 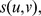 is defined by
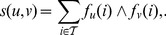
(13)


The rationale for 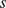 is that it generalizes the size of set intersections. If 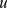 and 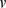 are each associated with a single evidence, 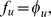 and 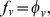 (13) becomes
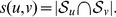
(14)


In general, the weights of proteins can vary significantly, both within and between complexes. Hence, it is more robust to compare the weight of each protein relative to the total weights of complexes (self-similarities), rather than absolutely. Define the relative distance measure 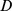 between complexes 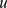 and 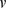 by
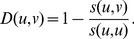
(15)


Clearly, 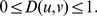 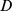 is an asymmetric distance measure, and 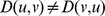 unless 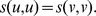 Note that 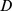 does not satisfy the triangle inequality. By construction of 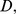 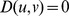 is equivalent to 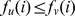 for each protein 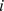 (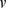 dominates 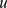). If all identical complexes are treated as one unit, it can be shown that the relation 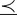 between complexes, defined by 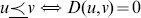 is a partial order. That is, the binary relation 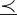 on the set of all complexes is reflexive, asymmetric and transitive. Every partial order naturally corresponds to a transitively closed DAG, where two elements are linked by a directed edge if they are related under the partial order.

#### Relationship graph

Although the partial order 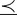 provides a desirable mathematical structure for investigating sub-part relationships, it is not sufficiently flexible to be directly applied to experimentally determined protein complexes. From a practical point of view, it is appropriate to consider 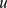 a subunit or a subcomplex of 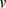 even when 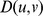 is greater than zero but still small. For example, due to experimental uncertainties, 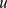 might be totally covered by 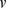 except for a single protein with a low weight. Given a set of complexes 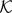 and a parameter 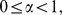 we construct a directed graph 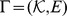 where
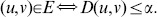
(16)


The graph 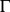 captures approximate part-of relationships between complexes. For 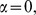 it corresponds to the partial order 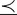 and hence is a transitively closed DAG. For 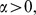 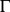 need not be transitively closed and it may contain cycles. In particular, there may be cases where both 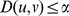 and 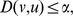 leading to a double (bi-directional) edge between 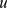 and 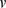 in 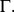 However, Proposition 1 below indicates that 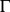 is always acyclic if it does not have bi-directional edges.

**Proposition 1.** *Let* 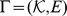 *be a directed graph where for* 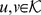 *,* 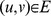 *if and only if* 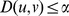 *for some* 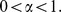 *Suppose that for any* 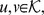 ** 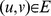 *implies* 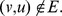 *Then,* 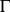 *contains no cycles.*

*Proof.* Let 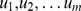 be a sequence of points in 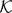 such that 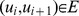 for all 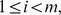 and suppose that it forms a cycle, that is, 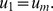 By our assumption, for all 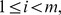 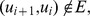 implying 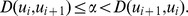 Hence, by (15), 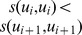 for all 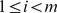 implying 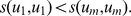 Therefore, 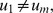 contradicting the claim that 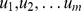 forms a cycle.

#### Iterative clustering of similar complexes

For small 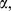 it is reasonable to treat two complexes 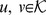 such that 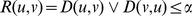 as equivalent, that is, as representatives of the same biological entity. Clustering such equivalent evidences for complexes produces a more informative hierarchy and at the same time leads to an acyclic graph by Proposition 1. To ensure that no equivalent complexes up to 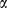 remain, we employ an iterative heuristic procedure that resembles hierarchical (agglomerative) clustering. Each protein complex node in the initial set 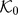 consists of all records sharing the same publication, experimental method and bait. The first clustering step involves merging all initial complexes that have identical support weights to form the *non-redundant set* 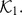 At each subsequent step, all pairwise distances between any two nodes are computed (or transferred from the previous step), and the two closest complexes according to the symmetrized distance 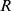 are replaced with a single node associated with the union of their evidences. Aggregating evidence sets 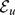 and 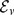 changes the weights of evidences in 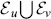, hence changing the adjusted weights and information flow weights of proteins belonging to 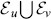. Therefore, for complexes containing proteins within 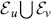, their mutual distances and their distances to all other nodes are now affected. Consequently, all the affected pairwise distances need to be re-computed prior to next aggregation. The procedure terminates when no pairwise 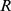-distances smaller than 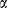 remain.

### Interpretation of complex hierarchies

#### Filtering complexes with small effective size

Since this investigation focuses on the large heteromeric complexes, the initial PC set includes only the evidences for complexes containing at least four proteins. However, using adjusted and information flow weights allows us to identify nominally large complexes that contain many of the common proteins and hence have a small effective size. For example, a four-subunit complex may contain three very common chaperones and one other protein and hence behave like a single-member complex with respect to the distance 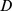 used to construct the hierarchy. To remove spurious links within the hierarchy, it is desirable to remove from consideration such nodes prior to further analysis. For a complex node 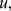 we measure its effective size using the *participation ratio* 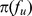 [31] of its weight function 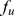


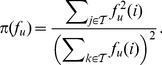
(17)


All nodes with the participation ratio (effective size) smaller than 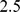 were removed from each hierarchy and considered separately. A complex of effective size smaller that 2.5 reflects more of the nature of binary interactions or a single protein.

#### Comparison of final DAGs constructed using different weighting schemes

The hierarchies obtained using our algorithm above contain different nodes and edges depending on the type of the weight function used and the value of 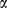. Hence, we compare DAGs from the same species by converting them to directed graphs over the non-redundant set 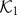, which is independent of the weight function used. Any merged complex node 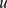 is expanded into its constituent non-redundant nodes 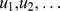, that are all linked pairwise via directed links in both directions. Each link 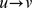 in the final hierarchy is expanded into links 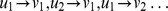 between the constituent non-redundant nodes. Then, comparison between two directed graphs 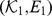 and 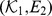 reduces to the comparison of the edge sets 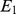 and 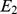 to discover matching and mismatching edges.

#### Enrichment analysis of complexes and components

We ascribed biological interpretation to the individual complexes and connected components of the constructed hierarchies through functional enrichment analysis based on the Gene Ontology (GO) [32], [33]. Each GO term annotates one or more proteins with a description of a biological process, cellular location or molecular function. To compute enrichment statistics, we used SaddleSum [34], [35], a tool we developed earlier. Given a set of weights over proteins, SaddleSum scores each GO term with the sum of the weights corresponding to its annotated proteins. It then estimates, depending on the number of genes involved, the P-value for that score by using the saddlepoint approximation [36] to the empirical distribution function derived from all weights. We consider a term significant if the E-value (the Bonferroni corrected P-value) for its score is smaller than 0.01. When we considered individual complexes (DAG nodes), we assigned to each protein its support weight. When we considered connected components (sets of nodes), we assigned to each protein the average support weight over all nodes within the component. Using weights allows us to take into account the representation of each protein within each node or component so that the proteins that occur in relatively few evidences have reduced influence on the enrichment results.

## Results

### Initial datasets

Using the procedure described above, we first extracted from a recent edition of iRefIndex the datasets of protein complexes and direct interactions in human (*Homo sapiens*) and yeast (*Saccharomyces cerevisiae*). These two species account for the overwhelming majority of available evidences of multimeric protein complexes. The summary of initial data sets is shown in Table 1. Overall, the yeast dataset contains more evidences for complexes but fewer reporting publications. Strikingly, most of the evidences in yeast come from very few high-throughput publications [37]– (Table S1): the top ten most prolific publications in yeast account for 70% of reported complexes, in contrast to only 16% in human. The human dataset contains more participating proteins and more direct interactions, but the rise in binary interactions does not correspond to the rise in the number of proteins, making it sparser relative to yeast.

**Table 1:** Initial Datasets of Complexes and Direct Interactions.

Species	Complexes					Direct Interactions			
	evidences	unique	proteins	publications	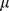	pairs	proteins	publications	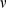
*S. cerevisiae*	9493	8754	4539	1560	57	20697	4950	3250	4
*H. sapiens*	7106	6153	7508	3366	17	34758	9451	11919	3

**Figure 2:**
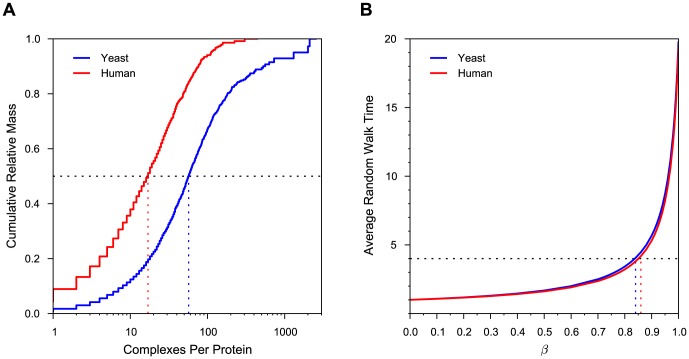
Estimating parameters for information flow weights from the initial datasets. **A** The parameter 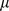 is defined as the weighted median number of complexes per protein: the proteins participating in more than 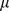 complexes account for 50% of the total ‘mass’ of all complexes. Here each protein member is assumed to contribute a single unit of mass to a complex. **B** The parameter 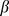 and the average time a randomly placed random walker spends in the protein-protein network are monotonically related. We set 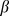 so that the average random walk time is exactly 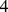 steps.

For protein complexes (PC set), the table shows the numbers of total evidences containing four or more proteins, compositionally unique (non-redundant) complexes, participating proteins and reporting publications. The parameter 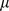 (see Methods and Fig. 2) is derived from the distribution of the number of complexes per protein. For direct interactions, the table shows the numbers of total pairs, participating proteins and reporting publications. Note that a publication may report both pull down experiments and direct interactions. The final column presents the parameter 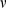, the median number of direct interactions per protein.

The most common proteins in yeast complexes (Table S2) include mostly chaperones, as well as cytoskeletal proteins (actin and tubulin), DNA helicase and a histone protein (HHF1). Due to their function, chaperones are naturally ‘sticky’ and associate with a large variety of proteins. Hence, it is not surprising that they would be found in many evidences for complexes. However, it should be noted that their prevalence is also a result of publication bias: the study of Gong *et al.* [37], the largest contributing publication for yeast, is exclusively concerned with chaperones. It uses all proteins as baits but only chaperones are considered as prey. The list of the most abundant proteins in human complexes (Table S3) is more varied. Apart from expected universal proteins such as chaperones and ubiquitin with associated ligases, it also contains the members of NuRD and SWI/SNF chromatin remodeling complexes, RNA polymerase, DNA ligases and helicases and the well studied p53 protein. Thus, the research bias is even more evident in the human dataset.

### Comparing protein weighting schemes

For both investigated species, we constructed three hierarchy DAGs using each of the three protein weighting schemes: support (code P), adjusted (code A), and information flow (code N4, where 4 indicates that *on average* four steps for random walks are allowed before termination). The parameter 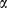 was set to 0.15, allowing embedding under support weights if approximately one seventh (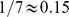) of the proteins or fewer do not match. Our choice of a noise tolerance level at 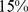 was inspired by the PageRank algorithm [42]. This algorithm, evaluating the importance of web pages, typically propagates 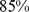 of each page's rank forward, indicating an intrinsic uncertainty at 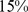 level, which we followed. Each DAG is referred to by concatenating an abbreviation of a species' name (sce for yeast, hsa for human) with a weighting scheme code. For example, the DAG based on the human dataset with supporting weights is labeled hsaP. In addition to these three types of DAGs, for each species we generated a DAG based on support weights and 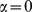 (code Z). This graph serves as a baseline for all three methods since its nodes are exactly the non-redundant (unique) complexes and each of its edges (with minor exceptions due to merging patterns) is contained in all other DAGs.

We estimated the parameters 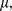 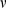 and 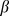 for the information flow weights from the initial datasets (Fig. 2). Due to abundance of common proteins in yeast and sparsity of the human dataset, the value of 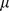 differs significantly between the two (57 against 17). However, the common proteins have comparable weights in both datasets (Tables S2 and S3). In both cases, a relatively small proportion of proteins participates in more than 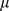 complexes and is thus down-weighted: 8.2% in yeast (344/4195) and 13.5% in human (918/6790). On the other hand, the value of the parameter 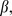 obtained by allowing on average four steps for random walks before termination, is very similar in both cases (0.84 for yeast and 0.85 for human). Although the networks of direct interactions in two species have very different global properties (Table 1), using the pseudocount parameter 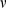 ensures that their curves in Fig. 2B are very close to each other.

**Figure 3:**
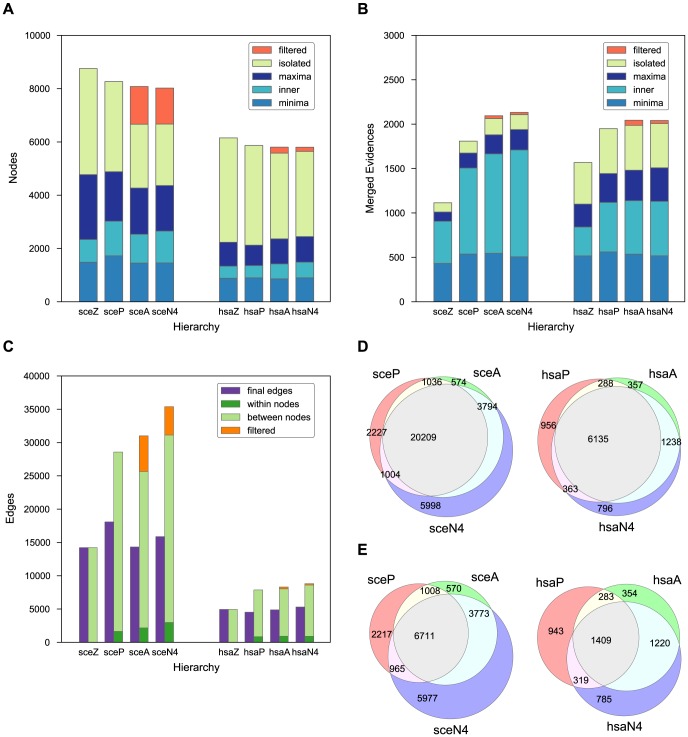
Comparison of complex hierarchies constructed using different protein weighting schemes. **A** Numbers of nodes. Each node is classified as either filtered (removed due to very small participation ratio), isolated (not connected with any other node), maximum (has only incoming edges), minimum (has only outgoing edges), and inner (has both incoming and outgoing edges). **B** Numbers of merged evidences. Each node contains one or more original evidences for a protein complex. This chart shows the breakdown of evidences that belong to nodes with more than one evidence, classified according to the type of the containing node. **C** Numbers of edges. For each DAG, shown are the numbers of total (left) and expanded (right) edges. Expanded edges are obtained by expanding each node into its constituent non-redundant complexes and linking them according to the links between the final nodes (see Methods). They are classified as ‘between nodes’, ‘within nodes’ or ‘filtered’ (either the source or the destination node are filtered). **D** Overlaps between expanded edges for P, A, and N4 schemes. **E** Overlaps between expanded edges for P, A, and N4 schemes, where the edges present in the baseline Z scheme are removed.

Overall, all three weighting schemes produce DAGs with similar numbers and types of nodes (Fig. 3A), with the main differences arising from using filtering based on participation ratios for A and N4 schemes, and from different merging patterns for similar nodes (Fig. 3B). The final DAGs contain significant numbers of isolated nodes, with a larger proportion in human. Of the nodes that are connected, the number of inner nodes is in all cases smaller than the number of either maxima (nodes with only incoming edges) or minima (nodes with only outgoing edges). However, many of the inner nodes contain merged identical or similar evidences (Fig. 3B).

**Figure 4:**
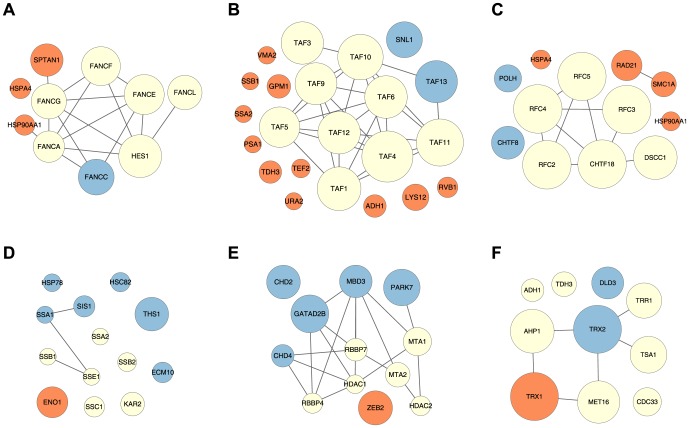
Examples of conflicting embeddings of complexes with respect to support and information flow weights. In each case a ‘smaller’ complex (X) embeds (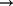) into a ‘larger’ one (Y) under one model but not the other. Each panel shows the union of proteins from both complexes linked by their direct interactions. Protein information flow weights are approximately indicated by the area of the corresponding nodes; the support weights of all proteins are equal. Proteins present only in X are colored red, those present only in Y are colored blue, while those belonging to both X and Y are displayed lightly colored. **Top row** (**A**, **B**, **C**) shows the cases where X 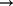 Y under information flow but not under support weights. **A** Members of the human Fanconi anaemia nuclear complex form a well-connected core of both X and Y. While Y contains an additional member of the same complex (FANCC), X includes two chaperones and one cytoskeletal protein that have significantly smaller weights than the shared core. Note that Y just fails to embed into X under 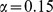 because the weights of the core proteins are somewhat larger in Y than in X due to interactions with FANCC not present in X. **B** Yeast TFIID complex is well-characterized through direct interactions. Hence, even though X contains 11/20 sticky proteins (mostly chaperones), it still embeds into Y. **C** Different manifestations of the human DNA replication factor C retrieved using the same bait protein. In this case both X 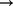 Y and Y 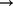 X, so X and Y would form a merged complex in the final hierarchy. **Bottom row** (**D**, **E**, **F**) shows the cases where X 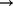 Y under support weights but not under information flow weights. **D** Gong *et al.* [37] systematically studied associations of yeast proteins with chaperones. In this case, X shares 6/7 proteins with B, which is sufficient for embedding under support weights. However, the one excluded protein (ENO1) is the bait for X, which carries relatively large information flow weight. **E** Members of the NuRD nuclear complex (light colored) are very common among evidences for human complexes and thus carry small information flow weights despite being well connected. Here, they appear as preys to transcription factor ZEB2 and multifunctional peptidase PARK7, which need not be very closely functionally related. **F** Yeast TRX1 and TRX2 are highly homologous isoenzymes that form a part of the cytoplasmic thioredoxin system. Although they share the same binding partners, they do not appear together in a complex and thus it is not appropriate to consider X a part of Y.

The number of edges varies more, particularly in yeast (Fig. 3C). When filtered and merged nodes are taken into account, the number of edges increases with the amount of information used (Z 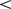 P 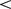 A 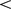 N4). The number of edges is in all cases much smaller in yeast than in human, both as an absolute number and with respect to the number of connected nodes. The sets of non-redundant edges, that is, induced relationships between non-redundant nodes, significantly overlap between P, A, and N4 schemes (Fig. 3C). However, most of the overlap is due to embeddings without any difference in protein composition, which are present in the Z scheme. When such cases are removed from consideration (Fig. 3D), it can be seen that while the overlap is still significant, the full information flow scheme (N4) provides a significant number of edges not established by other schemes. This effect is more pronounced in yeast than in human. However, there is also a non-negligible amount of relationships provided by the P scheme that do not hold under information flow weights. To qualitatively compare the differences in embeddings based on support and information flow weights, we investigated their conflicts on a case-by-case basis. Fig. 4 shows six selected examples drawn using Cytoscape [43]. Information flow scheme sharpens the cores of complexes by both strongly down-weighting extremely common members and by strengthening the ones linked through direct interactions (Fig. 4A–C). The latter feature distinguishes the proteins that are common but appear consistently together from the true ‘sticky’ ones, which occur almost randomly, and leads to relationships that cannot be established using adjusted weights. On the other hand, the majority of instances where embeddings occur under support but not under information flow weights, such as in Fig. 4D–F, arise between two functionally unrelated complexes. In yeast, many of such examples are from the Gong *et al.* [37] study, where two dissimilar bait proteins share the same set of chaperones (Fig. 4D). Another potentially noise-causing scenario shared by both yeast and human is when two different transcriptional regulators are attached to the same common machinery, such as the NuRD chromatin remodeling complex shown in Fig. 4E. By preventing establishment of relationships between such complexes, the information flow scheme distinguishes the ‘tool’ (a multifunctional part of the complex), from the basic functional core, which may be a single protein. The pattern from the examples in Fig. 4D–F, where the weight of the bait protein causes the embedding to fail under the N4 scheme, persists in a significant number of cases (1631/2217 in yeast, 299/943 in human).

### Topological features of the information flow hierarchies

Having shown in the previous section that the information flow weights (N4 scheme) provide the most extensive and biologically most sensible hierarchies, we will henceforth consider only sceN4 and hsaN4 as the representative hierarchies for yeast and human, respectively. Here, we broadly examine some topological features of these DAGs and their relation to biology.

#### Filtered and isolated nodes

As shown in Fig. 3A, the yeast hierarchy contains a non-negligible number (1351/8023) of nodes that are filtered due to very small effective size (participation ratio smaller than 2.5). Most filtered evidences (99%) originate from publications of Gong *et al.* [37], Gavin *et al.* [38] and Zhao *et al.* [44]. A typical example involves a bait protein with regular weight associated with a number of chaperones that are significantly down-weighted. In contrast, the number of filtered nodes in the human dataset is much smaller (157/5801) and no publication reports more than six filtered evidences. A very small number of removed nodes contains merged evidences (Fig. 3B).

Isolated nodes comprise 29% (2308/8023) of the entire hierarchy in yeast and 55% (3198/5801) in human. While some isolated nodes comprise merged evidences (Fig. 3B), the majority are associated with a single reported complex. The largest contributing publications provide most isolated nodes, both in yeast and in human. It is notable that the vast majority of evidences contributed by the largest human publications, such as Havugimana *et al.* [28] and Ewing *et al.* [45], belong to isolated nodes.

**Figure 5:**
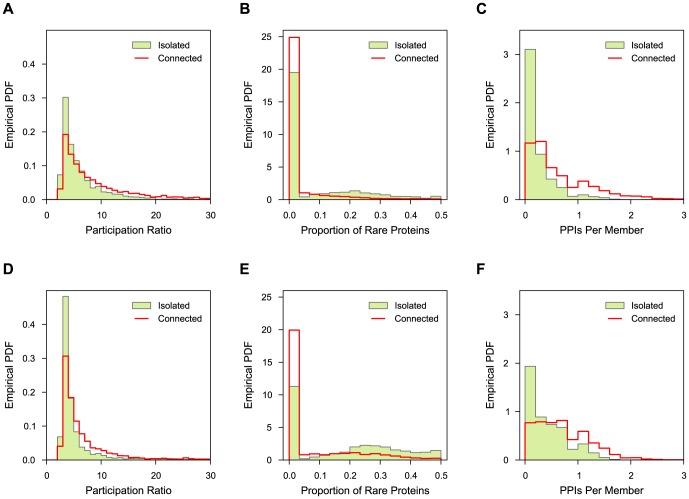
Comparison of empirical distributions of selected features of isolated and connected nodes. Each plot shows two histograms of empirical probability density functions for a node feature, one for isolated and one for connected nodes. Top row (**A**, **B**, **C**) shows features in yeast, bottom (**D**, **E**, **F**) in human. **Features:** participation ratio (effective complex size derived from protein weights –**A**, **D**), proportion of proteins occurring in not more than 4 complexes (**B**, **E**), and number of direct interactions per protein member (**C**, **F**).

To investigate whether a node's isolation can be explained by plausible simple criteria, we compared the distributions of participation ratios (effective complex sizes), proportions of infrequent proteins and numbers of directed interactions per protein for isolated and connected nodes in yeast and human (Fig. 5). While the distributions indeed slightly differ, it is clear that the isolated and connected groups cannot be separated using any single criterion.

#### Connected nodes

**Figure 6:**
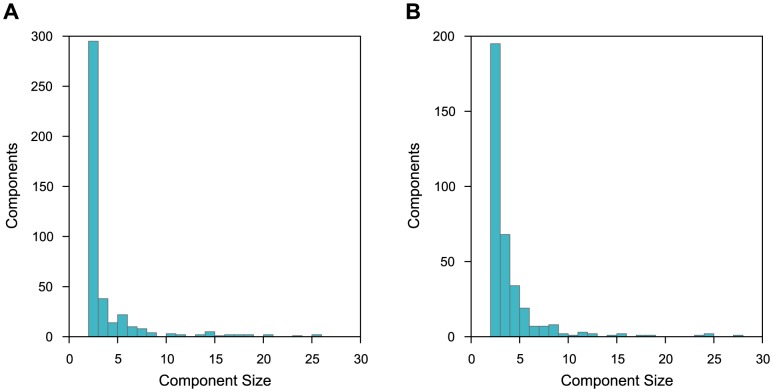
Histograms of sizes of connected components (in DAG nodes) for yeast (A) and human (B). Only the components with fewer than 30 nodes are shown. This excludes larger initial components (four in both yeast and human).

We found 4364/8023 connected nodes in yeast, and 2446/5801 in human. If the hierarchies are treated as undirected graphs, where two complex nodes are linked if either one is approximately a part of the other, they decompose into 420 connected components in yeast, and 360 in human. The distribution of component sizes is very nonuniform. The largest component contains 2807 nodes in yeast and 1031 in human, while the remaining ones mostly encompass very few nodes (Fig. 6).

A summary of the components with ten or more nodes in yeast and human are shown in Table S4 and S5, respectively. Based on enrichment results, it appears that in both species most components correspond either to a single complex or to few closely related complexes. The clear exceptions are the largest components in yeast and human, which cannot be characterized as a whole, except that they mostly contain nuclear proteins.

The connectivity pattern of the final hierarchy provides a way to describe the cellular compartmentalization of proteins from the complex data. Every protein generates a subgraph of the DAG, consisting of all nodes that contain the said protein. If the generated subgraph mostly consists of isolated nodes, it is very likely that the associations of the selected protein to these isolated nodes are due to noise. On the other hand, if the generated subgraph contains a relatively large set of related evidences, the selected protein may be viewed as an integral member of a persistent complex. This is often reflected by a relatively large set of connected nodes.

**Figure 7:**
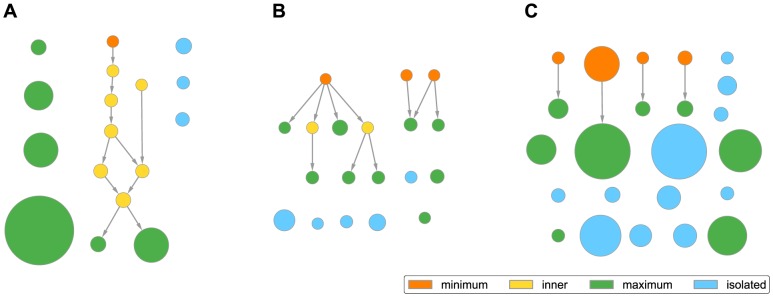
Examples of subgraphs of complex hierarchies induced by proteins. Each panel shows a subgraph of the entire hierarchy induced by the set of nodes containing a single selected protein. Each node's size indicates the participation ratio for the corresponding weight function, while its color indicates the topological type (see legend). An arrow linking two nodes indicates an approximately-part-of relationship. For clarity, only the ‘direct’ links are shown, while the links that could be replaced by a sequence of two or more direct links were removed. **A** Yeast ELP3 protein; **B** Human RPTOR protein; **C** Yeast GSY2 protein.

As an example, consider the subgraph of the yeast DAG induced by ELP3, a member protein of the transcriptional elongator complex (Fig. 7A). It consists of 17 nodes, 10 of which are connected together. The connectivity pattern of this component, with two chains converging towards a central node, indicates two separate assembly attracting centers that eventually merge into a stable core. Biologically, this suggests the existence of two distinct sub-complexes of the core elongator complex. Although this finding was first mentioned by Krogan and Greenblatt [46], it is strongly supported by numerous pull-down results from other publications in addition to theirs. The most central node of the component (the inner node linked to two maxima) represents the most comprehensive instance of the elongator complex. It contains eight proteins (IKI3, ELP2, ELP3, ELP4, IKI1, ELP6, KTI12, HRR25). Five out of seven nodes that are not connected to the main component contain exactly one of the two sub-complexes (IKI3, ELP2, ELP3). If three-member complexes were not filtered out from our dataset and this sub-complex was present, the connected component in this example would include most of the nodes containing ELP3.

The subgraph generated by human RPTOR protein (Fig. 7B) offers a different perspective. In this case, we observe two connected components and numerous isolated nodes, suggesting possible multiple biological functions of that protein. The GO annotation of RPTOR shows agreement with this observation. RPTOR is annotated as involved in the TOR signaling cascade, insulin receptor signaling pathway, cellular response to nutrient levels and cellular response to amino acid stimulus. We find that the larger component consists of complexes enriched for TOR signaling cascade, while the ones found in the smaller one are also enriched for insulin receptor signaling pathway. One of the isolated nodes is strongly enriched for cellular response to amino acid stimulus.

In contrast to the above two examples, the subgraph of the yeast GSY2 protein is almost completely disconnected. Given that the enzyme GSY2 is a glycogen synthase, which has an important metabolic role, this almost disconnected topological pattern is not surprising. In fact, we could not find any documented stable associations of GSY2.

## Discussion

An important step in our methodology is the filtering of complexes of small sizes that are most susceptible to accidental associations or false negatives. By restricting to complexes of size four or more (although effective size may be only 3), we are dropping some possibly noisy connections but do not lose the essential information. Coincidentally, this heuristic largely removes in yeast a large number of complexes, each made of chaperones and few other proteins, reported by Gong *et al.* in their high-throughput study concentrating on chaperone-protein interactions [37]. Another important step employed is to merge almost identical complexes. The reasons are twofold. First, due to nonuniformity in complex sampling, some complexes might be overly represented in comparison to others. This procedure allows one to merge overly represented but basically identical complexes to arrive at a more balanced representation. Second, the merging procedure effectively reduces the noise level because it down-weights occasional false protein associations and allows false negatives in one association data to be rescued by other nearly identical association data. However, we merge only complexes that are nearly equivalent but keep embedding relationships otherwise.

A DAG naturally emerges as a consequence of merging and embedding based on our similarity scoring. In this DAG construction, however, there are other important ingredients: downweighting frequently seen proteins, limiting the influence of proteins having few associations, and use of protein-protein interactions to emphasize proteins with documented binary interactions. Downweighting frequently binding proteins is not new [40], and this on its own is insufficient for our purpose. To avoid undue over emphasis on proteins with incomplete association and interaction information, two truncation parameters 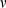 and 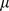 are introduced. To utilize independent information from PPIs, we also incorporated the ITM weights. The goal of ITM weights is to highlight the core proteins of each complex through *resonance* effect which amplifies weights of tightly connected sets of members. By considering only the walkers that both start and end at a complex 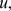 ITM weights measure the consistency of each complex, emphasizing proteins that are infrequent or that have many PPI links within the complex. The visits are scaled by 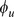 (support weight of a complex) to allow for different sizes of complexes.

Although PPIs play an important role in our DAG construction, it is worth pointing out that using PPI data alone may miss important information that only get revealed via association data. In Figure 4F, the two highly homologous isoenzymes TRX1 and TRX2 share the same binding partners. Therefore, these two proteins appear to be functional partners based on PPIs. The two complexes, headed by either TRX1 and TRX2 along with their interacting partners, are clearly distinct but carry the same function. However, TRX1 and TRX2 never appear together in a complex, indicating that it is inappropriate to treat them as members of the same complex. The only way one can faithfully retain the information that they never appear together is through association data. This shows that complexes complement binary interactions and properly combining them help in reducing unwanted noise.

We also note that, in the finally constructed DAG, there are many disconnected components. There are several possible reasons for this. Since we are pooling information from published data, the final collection is susceptible to sparsity of data, is dependent on different experimental/biological conditions, and may be biased towards tissue/disease-specific studies. Ideally, such less uniform samplings of complexes (protein associations) might be remedied by unbiased high-throughput approaches. Another way to mitigate the sparsity and bias in the human dataset is to use information obtained from mammalian models such as rat and mouse. This, however, requires appropriate functional mappings of orthologs, which is beyond the scope of the current study. As for the case of yeast DAG where many high-throughput data are employed, we still see many disconnected components. While this may simply suggest functional modularity, the largest components in both human and yeast DAGs, containing respectively more than 1000 and 2000 interconnected complexes, do not fit this picture. As shown in Table S4 and Table S5, these two largest components are mainly enriched for nuclear proteins and both evidently deserve further analyses.

The constructed DAGs can serve as a starting point (or a scaffold) for other algorithms for other computational analyses. Basing on published association data, these DAGs also form a knowledge database of complexes. Our DAG construction also offers a natural and well-balanced integration of complex data and binary data. By combining the DAGs with binary interaction data to form the underlying dataset for a network analysis tool, one can arrive at a stronger hypothesis-forming and exploration tool than that using only complexes data or binary data.

## Supporting Information

Figure S1 **Two procedures used by ppiTrim for complex deflation.**
(PDF)

Table S1 Top ten publications according to the number of reported complexes.
(PDF)

Table S2 Top 25 most frequent Yeast protein members of complexes.
(PDF)

Table S3 Top 25 most abundant Human protein members of complexes.
(PDF)

Table S4 Yeast components containing 10 or more nodes.
(PDF)

Table S5 Human components containing 10 or more nodes.
(PDF)

Text S1 **iRefIndex and ppiTrim.**
(PDF)

## References

[1] Yu H, Braun P, Yildirim MA, Lemmens I, Venkatesan K, et al (2008) High-quality binary protein interaction map of the yeast interactome network. Science 322: 104–110. 18719252 10.1126/science.1158684 PMC2746753

[2] Stellberger T, Hauser R, Baiker A, Pothineni VR, Haas J, et al (2010) Improving the yeast two-hybrid system with permutated fusions proteins: the Varicella Zoster Virus interactome. Proteome Sci 8: 8. 20205919 10.1186/1477-5956-8-8 PMC2832230

[3] Shimoda Y, Shinpo S, Kohara M, Nakamura Y, Tabata S, et al (2008) A large scale analysis of proteinprotein interactions in the nitrogen-fixing bacterium Mesorhizobium loti. DNA Res 15: 13–23. 18192278 10.1093/dnares/dsm028 PMC2650630

[4] Chien CT, Bartel PL, Sternglanz R, Fields S (1991) The two-hybrid system: a method to identify and clone genes for proteins that interact with a protein of interest. Proc Natl Acad Sci U S A 88: 9578–82. 1946372 10.1073/pnas.88.21.9578 PMC52761

[5] Legrain P, Selig L (2000) Genome-wide protein interaction maps using two-hybrid systems. FEBS Lett 480: 32–6. 10967325 10.1016/s0014-5793(00)01774-9

[6] Day RN, Periasamy A, Schaufele F (2001) Fluorescence resonance energy transfer microscopy of localized protein interactions in the living cell nucleus. Methods 25: 4–18. 11558993 10.1006/meth.2001.1211

[7] Havugimana PC, Wong P, Emili A (2007) Improved proteomic discovery by sample pre-fractionation using dual-column ion-exchange high performance liquid chromatography. J Chromatogr B Analyt Technol Biomed Life Sci 847: 54–61. 10.1016/j.jchromb.2006.10.075 17140863

[8] Qian WJ, Jacobs JM, Liu T, Camp DG 2nd, Smith RD (2006) Advances and challenges in liquid chromatography-mass spectrometry-based proteomics profiling for clinical applications. Mol Cell Proteomics 5: 1727–44. 16887931 10.1074/mcp.M600162-MCP200 PMC1781927

[9] Phizicky EM, Fields S (1995) Protein-protein interactions: methods for detection and analysis. Microbiol Rev 59: 94–123. 7708014 10.1128/mr.59.1.94-123.1995 PMC239356

[10] Rigaut G, Shevchenko A, Rutz B, Wilm M, Mann M, et al (1999) A generic protein purification method for protein complex characterization and proteome exploration. Nat Biotechnol 17: 1030–2. 10504710 10.1038/13732

[11] Puig O, Caspary F, Rigaut G, Rutz B, Bouveret E, et al (2001) The tandem affinity purification (tap) method: a general procedure of protein complex purification. Methods 24: 218–29. 11403571 10.1006/meth.2001.1183

[12] Alfarano C, Andrade CE, Anthony K, Bahroos N, Bajec M, et al (2005) The Biomolecular Interaction Network Database and related tools 2005 update. Nucleic Acids Res 33: D418–24. 15608229 10.1093/nar/gki051 PMC540005

[13] Isserlin R, El-Badrawi RA, Bader GD (2011) The Biomolecular Interaction Network Database in PSI-MI 2.5. Database (Oxford) 2011: baq037. 21233089 10.1093/database/baq037 PMC3021793

[14] Stark C, Breitkreutz BJ, Chatr-Aryamontri A, Boucher L, Oughtred R, et al (2011) The BioGRID interaction database: 2011 update. Nucleic Acids Res 39: D698–704. 21071413 10.1093/nar/gkq1116 PMC3013707

[15] Ruepp A, Waegele B, Lechner M, Brauner B, Dunger-Kaltenbach I, et al (2010) CORUM: the comprehensive resource of mammalian protein complexes–2009. Nucleic Acids Res 38: D497–501. 19884131 10.1093/nar/gkp914 PMC2808912

[16] Salwinski L, Miller CS, Smith AJ, Pettit FK, Bowie JU, et al (2004) The database of interacting proteins: 2004 update. Nucleic Acids Res 32: D449–51. 14681454 10.1093/nar/gkh086 PMC308820

[17] Keshava Prasad TS, Goel R, Kandasamy K, Keerthikumar S, Kumar S, et al (2009) Human Protein Reference Database – 2009 update. Nucleic Acids Res 37: D767–72. 18988627 10.1093/nar/gkn892 PMC2686490

[18] Aranda B, Achuthan P, Alam-Faruque Y, Armean I, Bridge A, et al (2010) The IntAct molecular interaction database in 2010. Nucleic Acids Res 38: D525–31. 19850723 10.1093/nar/gkp878 PMC2808934

[19] Ceol A, Chatr-Aryamontri A, Licata L, Peluso D, Briganti L, et al (2010) MINT, the molecular interaction database: 2009 update. Nucleic Acids Res 38: D532–9. 19897547 10.1093/nar/gkp983 PMC2808973

[20] Güldener U, Münsterkötter M, Oesterheld M, Pagel P, Ruepp A, et al (2006) MPact: the MIPS protein interaction resource on yeast. Nucleic Acids Res 34: D436–41. 16381906 10.1093/nar/gkj003 PMC1347366

[21] Pagel P, Kovac S, Oesterheld M, Brauner B, Dunger-Kaltenbach I, et al (2005) The MIPS mammalian protein-protein interaction database. Bioinformatics 21: 832–4. 15531608 10.1093/bioinformatics/bti115

[22] Brown KR, Jurisica I (2005) Online predicted human interaction database. Bioinformatics 21: 2076–82. 15657099 10.1093/bioinformatics/bti273

[23] Razick S, Magklaras G, Donaldson IM (2008) iRefIndex: a consolidated protein interaction database with provenance. BMC Bioinformatics 9: 405. 18823568 10.1186/1471-2105-9-405 PMC2573892

[24] Gillis J, Pavlidis P (2011) The impact of multifunctional genes on “guilt by association” analysis. PLoS ONE 6: e17258. 21364756 10.1371/journal.pone.0017258 PMC3041792

[25] Collins SR, Kemmeren P, Zhao XC, Greenblatt JF, Spencer F, et al (2007) Toward a comprehensive atlas of the physical interactome of saccharomyces cerevisiae. Mol Cell Proteomics 6: 439–50. 17200106 10.1074/mcp.M600381-MCP200

[26] Dutkowski J, Kramer M, Surma MA, Balakrishnan R, Cherry JM, et al (2013) A gene ontology inferred from molecular networks. Nat Biotechnol 31: 38–45. 23242164 10.1038/nbt.2463 PMC3654867

[27] Stojmirović A, Yu YK (2011) ppiTrim: constructing non-redundant and up-to-date interactomes. Database (Oxford) 2011: bar036. 21873645 10.1093/database/bar036 PMC3162744

[28] Havugimana PC, Hart GT, Nepusz T, Yang H, Turinsky AL, et al (2012) A census of human soluble protein complexes. Cell 150: 1068–81. 22939629 10.1016/j.cell.2012.08.011 PMC3477804

[29] Stojmirović A, Yu YK (2012) Information flow in interaction networks II: Channels, path lengths, and potentials. J Comput Biol 19: 379–403. 22409812 10.1089/cmb.2010.0228 PMC3317396

[30] Kemeny JG, Snell JL (1976) Finite Markov chains. New York: Springer-Verlag, ix+210 pp. Reprinting of the 1960 original, Undergraduate Texts in Mathematics.

[31] Stojmirović A, Yu YK (2007) Information flow in interaction networks. J Comput Biol 14: 1115–43. 17985991 10.1089/cmb.2007.0069

[32] Ashburner M, Ball CA, Blake JA, Botstein D, Butler H, et al (2000) Gene ontology: tool for the unification of biology. The Gene Ontology Consortium. Nat Genet 25: 25–29. 10802651 10.1038/75556 PMC3037419

[33] Gene Ontology Consortium (2010) The gene ontology in 2010: extensions and refinements. Nucleic Acids Res 38: D331–5. 19920128 10.1093/nar/gkp1018 PMC2808930

[34] Stojmirović A, Yu YK (2010) Robust and accurate data enrichment statistics via distribution function of sum of weights. Bioinformatics 26: 2752–9. 20826881 10.1093/bioinformatics/btq511 PMC2958744

[35] Stojmirović A, Bliskovsky A, Yu YK (2012) CytoSaddleSum: a functional enrichment analysis plugin for Cytoscape based on sum-of-weights scores. Bioinformatics 28: 893–4. 22345616 10.1093/bioinformatics/bts041 PMC3307116

[36] Lugannani R, Rice S (1980) Saddle point approximation for the distribution of the sum of independent random variables. Adv in Appl Probab 12: 475–490.

[37] Gong Y, Kakihara Y, Krogan N, Greenblatt J, Emili A, et al (2009) An atlas of chaperone-protein interactions in Saccharomyces cerevisiae: implications to protein folding pathways in the cell. Mol Syst Biol 5: 275. 19536198 10.1038/msb.2009.26 PMC2710862

[38] Gavin AC, Aloy P, Grandi P, Krause R, Boesche M, et al (2006) Proteome survey reveals modularity of the yeast cell machinery. Nature 440: 631–6. 16429126 10.1038/nature04532

[39] Krogan NJ, Cagney G, Yu H, Zhong G, Guo X, et al (2006) Global landscape of protein complexes in the yeast Saccharomyces cerevisiae. Nature 440: 637–43. 16554755 10.1038/nature04670

[40] Ho Y, Gruhler A, Heilbut A, Bader GD, Moore L, et al (2002) Systematic identification of protein complexes in Saccharomyces cerevisiae by mass spectrometry. Nature 415: 180–3. 11805837 10.1038/415180a

[41] Gavin AC, Bösche M, Krause R, Grandi P, Marzioch M, et al (2002) Functional organization of the yeast proteome by systematic analysis of protein complexes. Nature 415: 141–7. 11805826 10.1038/415141a

[42] Brin S, Page L (1998) The anatomy of a large-scale hypertextual web search engine. Comput Netw ISDN Syst 30: 107–117.

[43] Smoot ME, Ono K, Ruscheinski J, Wang PL, Ideker T (2011) Cytoscape 2.8: new features for data integration and network visualization. Bioinformatics 27: 431–2. 21149340 10.1093/bioinformatics/btq675 PMC3031041

[44] Zhao R, Davey M, Hsu YC, Kaplanek P, Tong A, et al (2005) Navigating the chaperone network: an integrative map of physical and genetic interactions mediated by the hsp90 chaperone. Cell 120: 715–27. 15766533 10.1016/j.cell.2004.12.024

[45] Ewing RM, Chu P, Elisma F, Li H, Taylor P, et al (2007) Large-scale mapping of human protein-protein interactions by mass spectrometry. Mol Syst Biol 3: 89. 17353931 10.1038/msb4100134 PMC1847948

[46] Krogan NJ, Greenblatt JF (2001) Characterization of a six-subunit holo-elongator complex required for the regulated expression of a group of genes in Saccharomyces cerevisiae. Mol Cell Biol 21: 8203–8212. 11689709 10.1128/MCB.21.23.8203-8212.2001 PMC99985

