# Supplementary material for: Building a Hierarchical Organization of Protein Complexes Out of Protein Association Data
Source: PLoS One. 2014 Jun 30;9(6):e100098. doi: 10.1371/journal.pone.0100098 (PMC4076247; doi:10.1371/journal.pone.0100098)
Supplement: Figure S1 — Two procedures used by ppiTrim for complex deflation. (PDF) [file pone.0100098.s001.pdf]

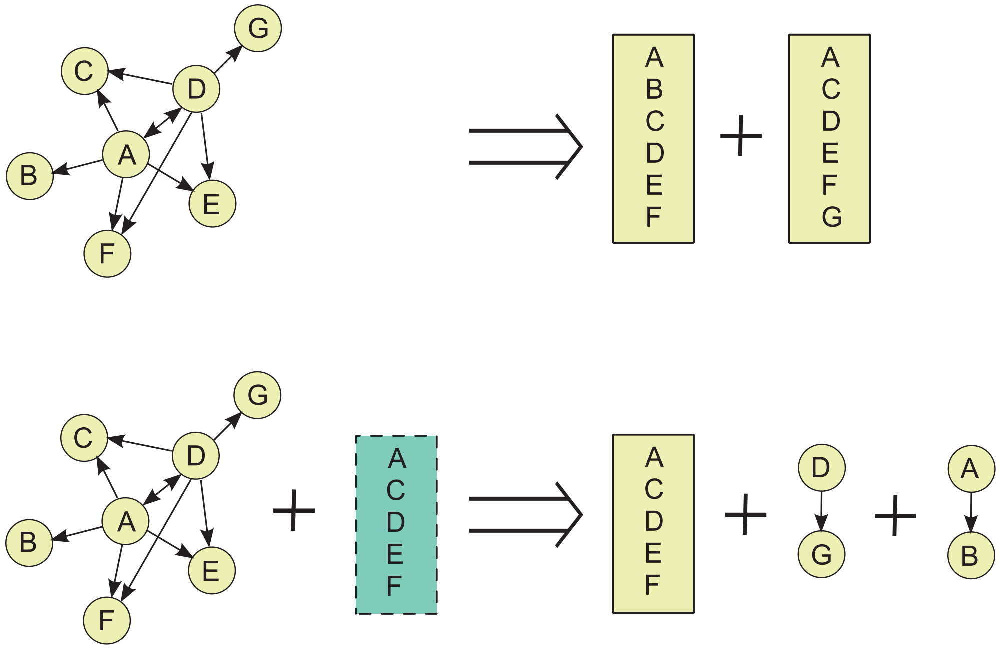

Figure S1. ppiTrim uses two procedures for complex deflation: pattern detection (top) and template matching (bottom). As an example, assume that a graph ABCDEFG, shown on the left, could be constructed from complex candidate interactions from a single publication. The arrow in A→B indicates a bait A to prey B relationship, while the interaction A↔D represents the bait-prey relationship twice, once with A and once with D as a bait. Pattern detection algorithm (top) recognizes A and D as hubs of potentially spoke-expanded complexes and thus replaces all pairwise interactions on the left with complexes ABCDEF and ACDEFG. Suppose that the complex ACDEF was reported from the same publication by a different database. Then, template matching procedure (bottom) would generate the complex ACDEF (with all other annotation, such as experimental detection method, retained from the original interactions) and remove all original interactions except D→G and A→B.
