# Supplementary material for: Building a Hierarchical Organization of Protein Complexes Out of Protein Association Data
Source: PLoS One. 2014 Jun 30;9(6):e100098. doi: 10.1371/journal.pone.0100098 (PMC4076247; doi:10.1371/journal.pone.0100098)
Supplement: Table S1 — Top ten publications according to the number of reported complexes. (PDF) [file pone.0100098.s002.pdf]

Table S1. Top Ten Publications According To Reported Complexes

Authors	Year	Pubmed ID	Evidences
Gong *et al.*	2009	19536198	1996 (21.0%)
Gavin *et al.*	2006	16429126	1557 (16.4%)
Krogan *et al.*	2006	16554755	1175 (12.4%)
Ho *et al.*	2002	11805837	906 (9.5%)
Gavin *et al.*	2002	11805826	461 (4.9%)
Breitkreutz *et al.*	2010	20489023	174 (1.8%)
Krogan *et al.*	2004	14759368	101 (1.1%)
Hermjakob *et al.*	2004	14681455	99 (1.0%)
Zhao *et al.*	2005	15766533	67 (0.7%)
Lambert *et al.*	2010	21179020	64 (0.7%)
TOTAL Top 10 – yeast			6600 (69.5%)

Authors	Year	Pubmed ID	Evidences
Havugimana *et al.*	2012	22939629	472 (6.6%)
Ewing *et al.*	2007	17353931	217 (3.1%)
Sowa *et al.*	2009	19615732	92 (1.3%)
Barrios-Rodiles *et al.*	2005	15761153	78 (1.1%)
Behrends *et al.*	2010	20562859	60 (0.8%)
Bouwmeester *et al.*	2004	14743216	53 (0.7%)
Li *et al.*	2011	21903422	48 (0.7%)
Hutchins *et al.*	2010	20360068	30 (0.4%)
Jeronimo *et al.*	2007	17643375	25 (0.4%)
Holaska & Wilson	2007	17620012	23 (0.3%)
TOTAL Top 10 – human			1098 (15.5%)

The table on the left shows the top publications reporting yeast complexes, while that on the right shows the main publications contributing to the human dataset. Each row provides the authors, year and Pubmed ID associated with a publication, as well as the counts of protein complexes (containing four or more proteins) reported, as an absolute number and as a perecentage of the entire dataset.
