# Supplementary material for: Building a Hierarchical Organization of Protein Complexes Out of Protein Association Data
Source: PLoS One. 2014 Jun 30;9(6):e100098. doi: 10.1371/journal.pone.0100098 (PMC4076247; doi:10.1371/journal.pone.0100098)
Supplement: Table S2 — Top 25 most frequent Yeast protein members of complexes. (PDF) [file pone.0100098.s003.pdf]

Table S2. Top 25 Most Frequent Yeast Protein Members of Complexes

Protein	Molecular Functions	Non-redundant Complexes	Initial Weight
SSB1	ATPase activity; calmodulin binding; unfolded protein binding	2525	0.02
SSA2	ATP binding; ATPase activity; unfolded protein binding	2107	0.03
SSA1	ATPase activity; unfolded protein binding	2006	0.03
SSE1	ATP binding; adenyl-nucleotide exchange factor activity; peptide binding	1307	0.04
TEF2	GDP binding; GTP binding; translation elongation factor activity	729	0.08
HSP78	ATPase activity; misfolded protein binding	630	0.09
VMA2	proton-transporting ATPase activity, rotational mechanism	559	0.10
HSC82	ATPase activity; ATPase activity, coupled; unfolded protein binding	526	0.11
SSB2	ATPase activity; unfolded protein binding	503	0.11
SSC1	ATPase activity; enzyme regulator activity	421	0.14
URA2	aspartate carbamoyltransferase activity; carbamoyl-phosphate synthase (glutamine-hydrolyzing) activity	409	0.14
PSA1	mannose-1-phosphate guanylyltransferase activity	399	0.14
YDJ1	ATPase activator activity; unfolded protein binding	357	0.16
SIS1	unfolded protein binding	351	0.16
HSP60	ATPase activity; DNA replication origin binding; chaperone binding; single-stranded DNA binding; unfolded protein binding	338	0.17
TUB2	structural constituent of cytoskeleton	325	0.18
ECM10	*unfolded protein binding*	316	0.18
TUB1	structural constituent of cytoskeleton	310	0.18
HHF1	DNA binding	293	0.19
PFD1	unfolded protein binding	272	0.21
HSP42	unfolded protein binding	260	0.22
ACT1	structural constituent of cytoskeleton	245	0.23
HSP82	ATPase activity, coupled; unfolded protein binding	241	0.24
RVB2	ATP-dependent 3'-5' DNA helicase activity; ATP-dependent 5'-3' DNA helicase activity	239	0.24
SSZ1	unfolded protein binding	235	0.24

Each row corresponds to a protein and shows its gene symbol, the (non-automatically derived) GO terms annotating its molecular function, the number of containing non-redundant complexes and its initial adjusted weight. The adjusted weight is calculated after collecting all identical entries into non-redundant complexes, but before merging of any compositionally different complexes, using Eq. (3) from the main text. Here, we assume that each protein is fully present in its complex (i.e. $\phi_u(i) = 1$) and that $\mu = 57$ for yeast. Note that ECM10 has no non-automatically derived (evidence code IEA) molecular function GO term and hence we show its IEA terms *in italics*.
