# Supplementary material for: Building a Hierarchical Organization of Protein Complexes Out of Protein Association Data
Source: PLoS One. 2014 Jun 30;9(6):e100098. doi: 10.1371/journal.pone.0100098 (PMC4076247; doi:10.1371/journal.pone.0100098)
Supplement: Table S3 — Top 25 most abundant Human protein members of complexes. (PDF) [file pone.0100098.s004.pdf]

Table S3. Top 25 Most Abundant Human Protein Members of Complexes

Protein	Molecular Functions	Non-redundant Complexes	Initial Weight
UBC	protease binding; protein binding	441	0.04
HDAC1	RNA polymerase II transcription corepressor activity; activating transcription factor binding; core promoter binding; enzyme binding; histone deacetylase activity; histone deacetylase binding; identical protein binding; protein binding; protein deacetylase activity; sequence-specific DNA binding transcription factor activity; transcription factor binding	303	0.06
HDAC2	chromatin binding; enzyme binding; histone deacetylase activity; protein binding; protein deacetylase activity; sequence-specific DNA binding; transcription factor binding	224	0.08
SMARCA4	DNA-dependent ATPase activity; Tat protein binding; androgen receptor binding; histone acetyl-lysine binding; p53 binding; protein N-terminus binding; protein binding; transcription coactivator activity; transcription corepressor activity	158	0.11
RBBP4	DNA-dependent ATPase activity; histone binding; histone deacetylase binding; protein binding	157	0.11
SIN3A	protein binding; transcription corepressor activity	150	0.11
CUL1	protein binding	147	0.12
HSPA8	ATPase activity, coupled; protein binding	138	0.12
SMARCC1	protein N-terminus binding; protein binding; transcription coactivator activity	133	0.13
HSP90AA1	ATP binding; ATPase activity; TPR domain binding; identical protein binding; nitric-oxide synthase regulator activity; nucleotide binding; protein binding; protein homodimerization activity	133	0.13
POLR2A	DNA binding; DNA-directed RNA polymerase activity; identical protein binding; protein binding; protein kinase activity; ubiquitin protein ligase binding	127	0.13
HSPA4	ATP binding	126	0.13
RUVBL2	ATP-dependent DNA helicase activity; ATPase activity; DNA helicase activity; identical protein binding; protein binding; unfolded protein binding	120	0.14
RUVBL1	DNA helicase activity; protein binding	118	0.14
TP53	ATP binding; DNA binding; RNA polymerase II transcription factor binding; RNA polymerase II transcription regulatory region sequence-specific DNA binding transcription factor activity involved in positive regulation of transcription; chaperone binding; chromatin binding; copper ion binding; damaged DNA binding; enzyme binding; histone acetyltransferase binding; identical protein binding; p53 binding; protease binding; protein N-terminus binding; protein binding; protein heterodimerization activity; protein kinase binding; protein phosphatase 2A binding; sequence-specific DNA binding transcription factor activity; transcription factor binding; transcription regulatory region DNA binding; ubiquitin protein ligase binding; zinc ion binding	115	0.15
SKP1	protein binding; ubiquitin-protein ligase activity	113	0.15
RBX1	NEDD8 ligase activity; protein binding; ubiquitin protein ligase binding; ubiquitin-protein ligase activity	113	0.15
SMARCB1	Tat protein binding; p53 binding; protein binding; transcription coactivator activity	111	0.15
PRKDC	DNA-dependent protein kinase activity; identical protein binding; protein binding; protein kinase activity; protein serine/threonine kinase activity; transcription factor binding	111	0.15
RBBP7	protein binding	110	0.15
SMARCC2	protein binding; transcription coactivator activity	108	0.16
ACTL6A	protein binding; transcription coactivator activity	108	0.16
PARP1	NAD+ ADP-ribosyltransferase activity; identical protein binding; protein N-terminus binding; protein binding; transcription factor binding	99	0.17
TRRAP	protein binding; transcription cofactor activity	98	0.17
XRCC6	5’-deoxyribose-5-phosphate lyase activity; DNA binding; double-stranded DNA binding; double-stranded telomeric DNA binding; identical protein binding; protein C-terminus binding; protein binding; transcription regulatory region DNA binding	91	0.19

Each row corresponds to a protein and shows its gene symbol, the (non-automatically derived) GO terms annotating its molecular function, the number of containing non-redundant complexes and its initial adjusted weight. The adjusted weight is calculated after collecting all identical entries into non-redundant complexes, but before merging of any compositionally different complexes, using Eq. (3) from the main text. Here, we assume that each protein is fully present in its complex (i.e. $\phi_u(i) = 1$) and that $\mu = 17$ for human.
