# Supplementary material for: Building a Hierarchical Organization of Protein Complexes Out of Protein Association Data
Source: PLoS One. 2014 Jun 30;9(6):e100098. doi: 10.1371/journal.pone.0100098 (PMC4076247; doi:10.1371/journal.pone.0100098)
Supplement: Table S4 — Yeast components containing 10 or more nodes. (PDF) [file pone.0100098.s005.pdf]

Table S4. Yeast Components Containing 10 or More Nodes

Component	Nodes				Gene Ontology Cellular Component
	Total	Minima	Inner	Maxima	
K000	2807	790	935	1082	macromolecular complex; nuclear part; nuclear lumen; nucleus; ribonucleoprotein complex; intracellular organelle part; nuclear DNA-directed RNA polymerase complex; intracellular non-membrane-bounded organelle; cytosolic part; proteasome complex; proteasome regulatory particle
K001	51	16	23	12	exosome (RNase complex); nuclear exosome (RNase complex); cytoplasmic exosome (RNase complex); nuclear lumen; nuclear part; nucleolus
K002	41	15	12	14	ER ubiquitin ligase complex; endoplasmic reticulum membrane; Cdc48p-Npl4p-Ufd1p AAA ATPase complex; Cdc48p-Npl4p-Vms1p AAA ATPase complex
K003	38	17	16	5	HOPS complex; CORVET complex; fungal-type vacuole membrane; SNARE complex
K004	31	16	8	7	mitochondrial small ribosomal subunit
K005	25	8	11	6	mitochondrial inner membrane presequence translocase complex; mitochondrial membrane part; presequence translocase-associated import motor
K006	25	8	15	2	anaphase-promoting complex
K007	23	9	8	6	exocyst; mating projection tip; cellular bud tip; incipient cellular bud site; site of polarized growth; cellular bud neck
K008	20	11	8	1	Golgi transport complex
K009	20	8	11	1	ribonuclease MRP complex; nucleolar ribonuclease P complex
K010	18	5	8	5	mitochondrial large ribosomal subunit
K011	18	6	4	8	site of polarized growth; actin cytoskeleton; actin filament; cytoskeleton; cellular bud tip; cytoskeletal part; myosin complex; cortical actin cytoskeleton
K012	17	8	7	2	TRAPP complex; trans-Golgi network
K013	17	8	2	7	SNARE complex; Dsl1p complex; endoplasmic reticulum
K014	16	4	8	4	oligosaccharyltransferase complex
K015	16	8	5	3	mitochondrial respiratory chain; respiratory chain; mitochondrial membrane part; mitochondrial respiratory chain complex IV; mitochondrial respiratory chain complex III; integral to membrane
K016	15	4	2	9	ER to Golgi transport vesicle
K017	14	5	4	5	condensed nuclear chromosome; condensed chromosome; nuclear condensin complex; nuclear chromosome part; chromosomal part; chromosome passenger complex; spindle midzone; CBF3 complex; intracellular non-membrane-bounded organelle; kinetochore; condensed nuclear chromosome kinetochore
K018	14	2	8	4	Set1C/COMPASS complex
K019	14	5	7	2	DASH complex; spindle; microtubule cytoskeleton; cytoskeletal part; spindle microtubule

Component	Nodes				Gene Ontology Cellular Component
	Total	Minima	Inner	Maxima	
K020	14	2	6	6	proton-transporting ATP synthase complex; mitochondrial proton-transporting ATP synthase complex; proton-transporting two-sector ATPase complex; mitochondrial proton-transporting ATP synthase complex, coupling factor F(o); proton-transporting ATP synthase complex, coupling factor F(o); mitochondrial membrane part; proton-transporting ATP synthase complex, catalytic core F(1); mitochondrial proton-transporting ATP synthase complex, catalytic core F(1); mitochondrial proton-transporting ATP synthase, stator stalk; mitochondrion; mitochondrial proton-transporting ATP synthase, central stalk; integral to membrane
K021	14	6	3	5	alpha-1,6-mannosyltransferase complex
K022	13	6	3	4	SNARE complex; ER to Golgi transport vesicle
K023	13	5	4	4	cyclin-dependent protein kinase holoenzyme complex
K024	11	4	3	4	nuclear mitotic cohesin complex
K025	11	4	6	1	endoplasmic reticulum
K026	10	3	5	2	peroxisomal membrane
K027	10	4	1	5	carboxy-terminal domain protein kinase complex
K028	10	1	6	3	GID complex

For each connected component, the columns give its ID, the total number of nodes contained, the numbers of minima, inner nodes and maxima, and the most significant Gene Ontology terms for cellular component that were associated with it using enrichment analysis.
