# Supplementary material for: Building a Hierarchical Organization of Protein Complexes Out of Protein Association Data
Source: PLoS One. 2014 Jun 30;9(6):e100098. doi: 10.1371/journal.pone.0100098 (PMC4076247; doi:10.1371/journal.pone.0100098)
Supplement: Table S5 — Human components containing 10 or more nodes. (PDF) [file pone.0100098.s006.pdf]

Table S5. Human Components Containing 10 or More Nodes

Component	Nodes				Gene Ontology Cellular Component
	Total	Minima	Inner	Maxima	
K000	1031	315	365	351	nucleoplasm; nuclear part; nuclear lumen; macromolecular complex; nucleus; intracellular organelle part; chromosome; histone methyltransferase complex; proteasome complex; ribonucleoprotein complex; Ino80 complex; intracellular part
K001	53	16	28	9	Fanconi anaemia nuclear complex
K002	46	15	17	14	SAGA-type complex; transcription factor TFTC complex; DNA-directed RNA polymerase II, holoenzyme; transcription factor TFIID complex; nucleoplasm part; MLL1 complex
K003	38	13	20	5	PcG protein complex
K004	36	14	15	7	anaphase-promoting complex; nucleoplasm
K005	27	10	11	6	death-inducing signaling complex
K006	24	7	7	10	Cdc73/Paf1 complex
K007	24	12	1	11	AMP-activated protein kinase complex
K008	23	5	1	17	SMAD protein complex
K009	18	2	7	9	ESC/E(Z) complex
K010	17	6	6	5	BBSome; microtubule basal body; motile cilium
K011	15	6	4	5	BRCA1-A complex; BRISC complex; nuclear ubiquitin ligase complex
K012	15	4	6	5	transcriptional repressor complex; histone deacetylase complex; spindle microtubule
K013	14	7	2	5	-
K014	12	1	0	11	histone deacetylase complex; transcriptional repressor complex
K015	12	3	2	7	-
K016	11	5	4	2	replication fork; nuclear replication fork; nuclear replisome; DNA polymerase complex; nuclear chromosome part; nucleoplasm; nucleotide-excision repair complex
K017	11	3	5	3	-
K018	11	7	3	1	mitochondrial respiratory chain complex I
K019	10	1	1	8	endosome membrane

For each connected component, the columns give its ID, the total number of nodes contained, the numbers of minima, inner nodes and maxima, and the most significant Gene Ontology terms for cellular component that were associated with it using enrichment analysis. Components K013, K015, and K017 have no significant Gene Ontology terms for cellular component with E-value smaller than our cutoff 10^{-3}.
