# Supplementary material for: Building a Hierarchical Organization of Protein Complexes Out of Protein Association Data
Source: PLoS One. 2014 Jun 30;9(6):e100098. doi: 10.1371/journal.pone.0100098 (PMC4076247; doi:10.1371/journal.pone.0100098)
Supplement: Text S1 — iRefIndex and ppiTrim. (PDF) [file pone.0100098.s007.pdf]

Text S1: iRefIndex and ppiTrim

iRefIndex [1] is a comprehensive and consistent index of protein interactions available in a number of primary interaction databases such as BIND [2, 3], BioGRID [4], CORUM [5], DIP [6], HPRD [7], IntAct [8], MINT [9], MPact [10], MPPI [11] and OPHID [12]. Apart from collecting the protein interactions from a variety of sources into a single dataset, iRefIndex addresses the problem of inconsistent protein identifiers used by different source databases by mapping the sequence of every interactor into a unique identifier. A major goal for iRefIndex is to maintain the provenance of each interaction record, enabling unambiguous identification of its original source. This approach, however, has a disadvantage that the interactions from an experiment annotated by multiple databases appear as distinct records, sometimes with apparently conflicting annotations. Furthermore, protein complexes are represented inconsistently across source databases: some sources represent complexes as flat lists of proteins, while other expand them into binary interactions using a spoke or matrix model [13].

To obtain non-redundant sets of protein interactions and complexes, we have earlier developed a script ppiTrim [14], which consolidates iRefIndex records so that each experimental evidence for an interaction or association appears exactly once. ppiTrim proceeds in three stages: mapping all interactors to gene identifiers, deflating potentially expanded complexes, and reconciling for each interaction the annotation labels among different source databases. It outputs three broad classes of interactions: directed binary interactions (biochemical reactions with asymmetric biological roles of interactors), undirected binary interactions (physical bindings), and protein complexes (associations of more than two proteins). For the current study, we developed version 1.3 of ppiTrim (available at `http://www.ncbi.nlm.nih.gov/CBBresearch/Yu/downloads/ppiTrim.html`), which is able to parse the iRefIndex release 10.0. ppiTrim-1.3 also differs from ppiTrim-1.1 in additional details, which we describe below.

At its second stage, ppiTrim attempts to detect and replace spoke-expanded complexes from pull-down experiments with flat lists of proteins. The algorithm operates on sets of interaction records grouped by publication and source database, using two procedures for consolidation: pattern detection and template matching (Fig. S1). Pattern detection relies on annotations of experimental roles for proteins as baits or preys: a bait linked to several preys is converted into a protein list. Template matching involves matching a group of candidate interactions from one source database to the complexes (lists) indicated by other databases. ppiTrim-1.1 used the pattern detection algorithm only on sets of interactions from the BioGRID and template matching on all sets, including those originating from the BioGRID. This could result in several, slightly different, representations of the same complex from

a publication. The algorithm employed by ppiTrim-1.3 uses pattern detection on all sets of interactions that have bait and prey labels for interactors, and template matching only for those sets where such information is missing.

The third stage of ppiTrim involves consolidation of all evidences for an interaction obtained from an experiment into a single record, and detection of potential annotation conflicts between different sources describing the same experiment. ppiTrim-1.1 only detects conflicts for binary interactions, while ppiTrim-1.3 adds a check for conflict between records representing complexes. It assumes that all records of complexes that share the same protein bait, experimental method annotation and publication describe the results of a single pull-down experiment, even if the lists of members do not fully agree. Such a situation arises when the same publication is curated by several source databases that disagree, or when a single source database reports results obtained using different protein detection methods, such as MALDI-TOF or MS/MS, as distinct records. Consolidating these records into one would lead to loss of information and hence ppiTrim-1.3 only labels them as being 'in conflict' with each other. In the current study, such groups of conflicting records are consolidated into a single entry of the initial PC set, where each protein member of a consolidated entry is associated with a support weight that is the average of the contributions from component records.

Supplementary References

S1. Razick S, Magklaras G, Donaldson IM (2008) iRefIndex: a consolidated protein interaction database with provenance. BMC Bioinformatics 9: 405.

S2. Alfarano C, Andrade CE, Anthony K, Bahroos N, Bajec M, et al. (2005) The Biomolecular Interaction Network Database and related tools 2005 update. Nucleic Acids Res 33: D418-24.

S3. Isserlin R, El-Badrawi RA, Bader GD (2011) The Biomolecular Interaction Network Database in PSI-MI 2.5. Database (Oxford) 2011: baq037.

S4. Stark C, Breitkreutz BJ, Chatr-Aryamontri A, Boucher L, Oughtred R, et al. (2011) The BioGRID interaction database: 2011 update. Nucleic Acids Res 39: D698-704.

S5. Ruepp A, Waegele B, Lechner M, Brauner B, Dunger-Kaltenbach I, et al. (2010) CORUM: the comprehensive resource of mammalian protein complexes–2009. Nucleic Acids Res 38: D497-501.

S6. Salwinski L, Miller CS, Smith AJ, Pettit FK, Bowie JU, et al. (2004) The database of interacting proteins: 2004 update. Nucleic Acids Res 32: D449-51.

S7. Keshava Prasad TS, Goel R, Kandasamy K, Keerthikumar S, Kumar S, et al. (2009) Human Protein Reference Database – 2009 update. Nucleic Acids Res 37: D767-72.

S8. Aranda B, Achuthan P, Alam-Faruque Y, Armean I, Bridge A, et al. (2010) The IntAct molecular interaction database in 2010. Nucleic Acids Res 38: D525-31.

S9. Ceol A, Chatr-Aryamontri A, Licata L, Peluso D, Briganti L, et al. (2010) MINT, the molecular interaction database: 2009 update. Nucleic Acids Res 38: D532-9.

S10. Güldener U, Münsterkötter M, Oesterheld M, Pagel P, Ruepp A, et al. (2006) MPact: the MIPS protein interaction resource on yeast. Nucleic Acids Res 34: D436-41.

S11. Pagel P, Kovac S, Oesterheld M, Brauner B, Dunger-Kaltenbach I, et al. (2005) The MIPS mammalian protein-protein interaction database. Bioinformatics 21: 832-4.

S12. Brown KR, Jurisica I (2005) Online predicted human interaction database. Bioinformatics 21: 2076-82.

S13. De Las Rivas J, Fontanillo C (2010) Protein-protein interactions essentials: key concepts to building and analyzing interactome networks. PLoS Comput Biol 6: e1000807.

S14. Stojmirović A, Yu YK (2011) ppiTrim: constructing non-redundant and up-to-date interactomes. Database (Oxford) 2011: bar036.
